# Supplementary material for: Rapid population decline in migratory shorebirds relying on Yellow Sea tidal mudflats as stopover sites
Source: Nat Commun. 2017 Apr 13;8:14895. doi: 10.1038/ncomms14895 (PMC5399291; doi:10.1038/ncomms14895)
Supplement: Supplementary Information — Supplementary Figures, Supplementary Tables and Supplementary Note [file ncomms14895-s1.pdf]

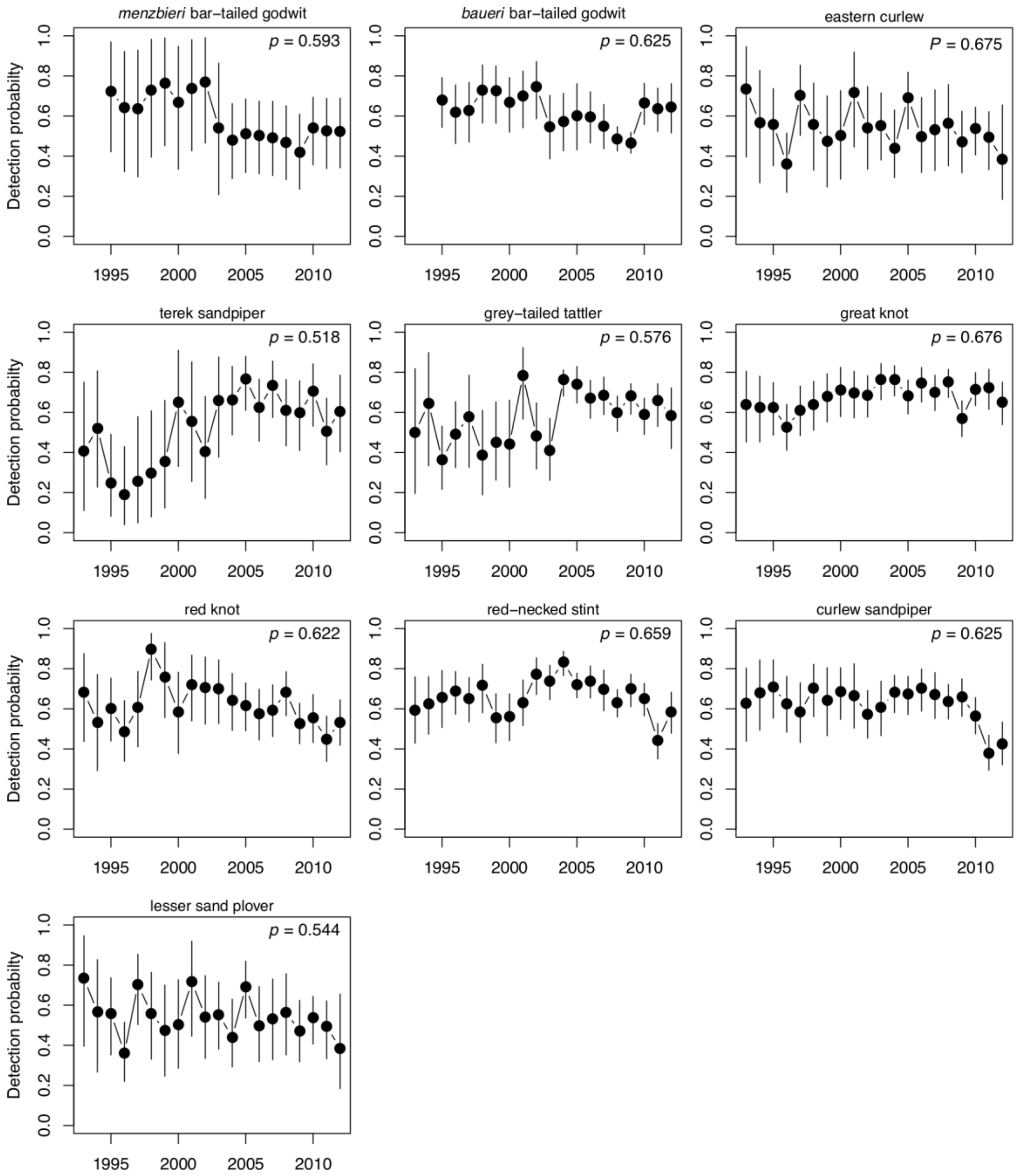

Supplementary Figure 1 | Detection probabilities from Bayesian N-mixture models of abundance from 1993-2012 for 10 EAAF migratory shorebird taxa. Points show posterior mean estimates of detection probability for each year of the time series. Error bars represent 95% credible intervals (CRI) around annual detection probability. The mean detection probability for the time series (p) appears in the upper right of each panel.

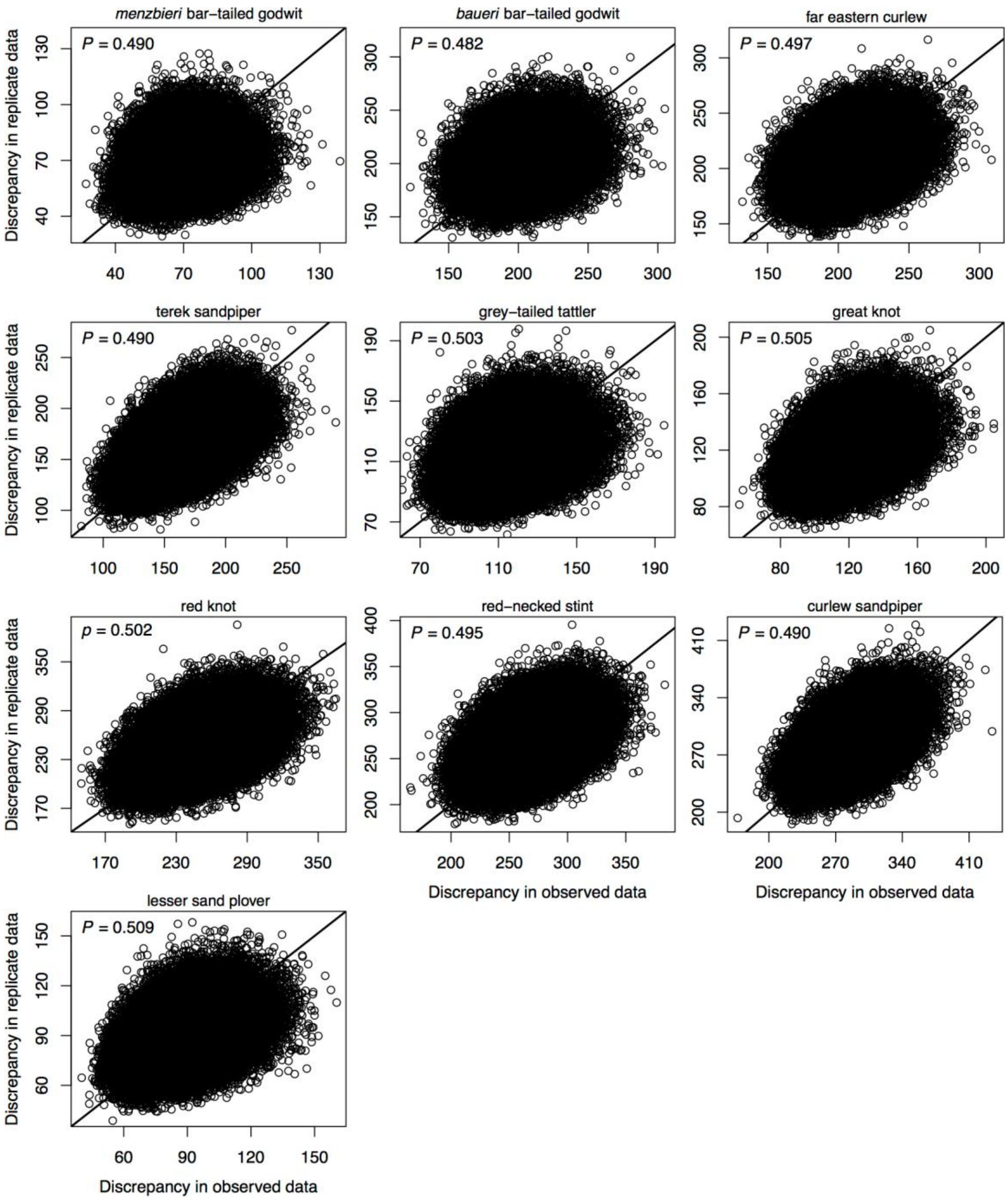

Supplementary Figure 2 | Posterior predictive checks for Bayesian N-mixture models of abundance from 1993-2012 for 10 EAAF shorebird taxa. Points represent the relationship between the discrepancies in the observed data and the discrepancies in replicate data simulated from the posterior distribution of 100,000 model iterations. Discrepancy is a chi-squared goodness of fit statistic. Solid lines show the 1:1 relationship in discrepancy between observed and replicated data. Bayesian *P*-values (*P*), the proportion of model iterations where discrepancies in the observed data exceed those of the replicate data, appear in the upper left of each panel. *P*-values should be near 0.50 when model fit is adequate.

Supplementary Table 1 | Spatial and temporal distribution of count data used to estimate abundance and population trends from 1993-2012 for 10 EAAF migratory shorebird taxa.

				Taxon									
Site	Node	Months counted	Roosts counted	*menzbieri* bar-tailed godwit	*baueri* bar-tailed godwit	far eastern curlew	Terek sandpiper	gray-tailed tattler	great knot	red knot	red-necked stint	curlew sandpiper	lesser sand plover
Albany Harbours	1	Jan-Mar	1						•	•	•		
Boucat Bay	1	Mar	NA						•				
Bush Point	1	Oct-Dec	1	•		•	•	•	•	•	•	•	
Chambers Bay	1	Dec	NA				•						
East Alligator Creek	1	Dec	NA				•						
Eighty Mile Beach	1	Oct-Dec	12	•		•	•	•	•	•	•	•	
Elcho Island	1	Nov-Dec	NA	•									
Fog Bay	1	Nov-Dec	NA				•		•				
Millingimbi Coast	1	Mar	NA	•			•						
Lake MacLeod	1	Sep-Nov, Jan, Mar	NA							•	•	•	
North Darwin	1	Nov-Feb	NA						•	•			•
Roebuck Bay	1	Oct-Dec	15	•		•	•	•	•	•	•	•	
Botany Bay	2	Nov-Feb	12			•		•			•	•	
Cairns Area	2	Jan-Feb	6			•		•			•	•	•
Great Sandy Strait	2	Jan-Feb	29		•	•	•	•			•	•	•
Hunter Estuary	2	Jan-Feb	15		•	•					•	•	•
Mackay	2	Nov-Feb	18			•	•	•	•		•		•
Moreton Bay	2	Jan-Feb	37		•	•	•	•	•	•	•	•	•
Swan Bay & Mud Islands	2	Jan-Feb	9			•			•	•	•	•	•
Townsville	2	Nov-Feb	1			•		•	•		•		•
Cape Portland	3	Nov-Feb	2								•	•	
Coorong	3	Nov-Feb	3								•	•	
Corner Inlet	3	Jan-Feb	27		•	•			•	•	•	•	
George Town	3	Nov-Feb	1			•					•	•	
Gulf of St. Vincent	3	Nov-Feb	12							•	•	•	
Lake Connewarre	3	Nov-Feb	6			•					•	•	
Laverton / Altona	3	Jan-Feb	7								•	•	
Moolap Saltworks	3	Jan-Feb	1								•	•	
Robbins Passage	3	Jan-Feb	10		•	•				•	•	•	
Southeast Tasmania	3	Jan-Feb	1			•					•	•	
Werribee / Avalon	3	Jan-Feb	1			•					•	•	
Western Port Bay	3	Nov-Feb	15			•				•	•	•	
Farewell Spit	4	Nov, Feb	NA		•					•			
Firth of Thames	4	Nov, Feb	NA		•					•			
Kaipara Harbour	4	Nov, Feb	NA		•					•			
Kawhia HArbour	4	Nov, Feb	NA		•								
Manukau Harbour	4	Nov, Feb	NA		•					•			
Ohiwa Harbour	4	Nov	NA		•								
Parengarenga Harbour	4	Nov, Feb	NA		•					•			
Rangaunu Harbour	4	Nov, Feb	NA		•					•			
Tasman Bay	4	Nov, Feb	NA		•								
Tauranga Harbour	4	Nov, Mar	NA		•								
Whangarei Harbour	4	Nov, Feb	NA		•					•			

A list of key contributors to the collection and management of these data is in the Acknowledgements and in the Supplementary Note 1.

Supplementary Table 2 | Population trend estimates and 95% credible intervals (CRI) from 1993-2012 for 8 EAAF migratory shorebird taxa across four non-breeding population nodes in Australia and New Zealand.

	Node			
Taxon	north Western Australia	Queensland	southeastern Australia	New Zealand
far eastern curlew	-0.023 (-0.065, 0.020)	**-0.052 (-0.069, -0.035)**	**-0.067 (-0.084, -0.061)**	–
curlew sandpiper	-0.019 (-0.088, 0.059)	**-0.075 (-0.098, -0.053)**	**-0.028 (-0.113, -0.052)**	–
great knot	**-0.057 (-0.096, -0.020)**	**-0.035 (-0.064, -0.004)**	–	–
red knot	-0.044 (-0.095, 0.006)	–	**-0.041 (-0.073, -0.007)**	**-0.042 (-0.069, -0.015)**
baueri bar-tailed godwit	–	–	-0.010 (-0.025, 0.004)	**-0.015 (-0.024, -0.005)**
Terek sandpiper	**-0.058 (-0.118, -0.001)**	0.006 (-0.043, 0.053)	–	–
red-necked stint	0.033 (-0.025, 0.086)	-0.013 (-0.030, 0.004)	0.018 (-0.010, 0.045)	
grey-tailed tattler	0.004 (-0.067, 0.072)	0.010 (-0.024, 0.042)	–	–

Boldface estimates indicate credibly declining populations.

Supplementary Table 3 | Pairwise odds ratios and 95% credible intervals (CRI) comparing population trends among non-breeding nodes for 8 EAAF shorebird taxa found on multiple nodes.

	Node comparison				
Taxon	1v2	1v3	1v4	2v3	3v4
far eastern curlew	1.03 (0.99, 1.08)	1.04 (1.00, 1.09)	–	1.01 (0.99, 1.04)	–
curlew sandpiper	1.06 (1.00, 1.14)	1.05 (1.00, 1.13)	–	1.01 (0.98, 1.04)	–
great knot	1.03 (0.98, 1.07)	–	–	–	–
red knot	–	1.00 (0.94, 1.06)	1.00 (0.95, 1.05)	–	1.00 (0.96, 1.04)
baueri bar-tailed godwit	–	–	–	–	1.01 (0.99, 1.02)
Terek sandpiper	1.06 (1.00, 1.15)	–	–	–	–
red-necked stint	1.01 (0.99, 1.07)	1.05 (0.99, 1.10)	–	1.04 (1.00,1.07)	–
grey-tailed tattler	1.00 (0.93, 1.08)	–	–	–	–

Lesser sand plover and the *menzbieri* subspecies of bar-tailed godwit were not included in these analyses because they occurred on only one node.

Supplementary Note 1 | List of key contributors to the shorebird datasets used in this paper.

Organizations.

Albany Bird Club, Atlas of Australian Birds, Broome Bird Observatory, Esperance Bird Observers Club, Eyre Bird Observatory, Global Flyway Network, New South Wales Wader Study Group, Sunraysia BOCA, Western Australia Wader Study Group, Shorebirds 2020 program.

Individuals.

David Adams, Jack Adams, Richard Alcorn, Laurel Allsopp, Peter Anton, Mark Antos, George Appleby, Richard Ashby, Rodney Attwood, George & Teresa Baker, Mike Bamford, Mark Barter, Pauline Bartels, Chris Baxter, Dawn Beck, Rod Bird, Stuart Blackhall, Matt Bloor, Anne Bondin, Jack & Pat Bourne, Adrian Boyle, Chris Brandis, Linda Brannian, Rob Breeden, Alan Briggs, Hazel Britton, Nigel and Mavis Burgess, Jeff Campbell, Graham Carpenter, Derek Carter, Mike Carter, Denis Charlesworth, Maureen Christie, Jane Cleary, Greg Clancy, Rohan Clarke, Simon Clayton, Jane Cleary, Rob Clemens, David Close, Chris Coleborn, Lisa Collins, Bob Cook, Jane Cooper, Ralph and Barbara Cooper, Ricki Coughlan, Trevor Cowie, Phil Craven, Liz Crawford, Linda Cross, Peter Dann, Chris Davey, Wendy Davies, Kieth Davis, Frank Day, Alma de Rebeira, Gay Deacon, Xenia Dennett, Dave Donato, Peter Driscoll, Peter Duckworth, Phil Du Guesclin, John Eckert, David Edmonds, Glenn Ehmke, Len Ezzy, Rob Farnes, Winston Filewood, Shirley Fish, Tony Flaherty, Alan Fletcher, Duncan Fraser, Barbara Garrett, Andrew Geering, Les George, Heather Gibbs, Alan Gillanders, Ken Gosbell, Doris Graham, Iva Graney, Travis Hague, Margaret Hamon, Ian Hance, Birgita Hansen, Sandra

Harding, Judy Harrington, Ken Harris, Chris Hassell, Rick Hawthorne, Jane Hayes, Bruce Haynes, Bryan and Toni Haywood, Colin Heap, Chris Herbert, Ash Herrod, Tony Hertog, Marilyn Hewish, Janice Hosking, Dean Ingwersen, Roger Jaensh, David James, Chris James, Roz Jessop, Penny Johns, Steve Johnson, Arthur and Sheryl Keates, Cill Kinross, Marcel Klaasen, Wally Klau, Peter Langdon, Jenny Lau, John Lauri, Sharon Lehman, Michael Lenz, Ann Lindsey, John Lowry, Richard Loyn, Hans Lutter, Grainne Maguire, Sue Mather, Bernie McCarrick, David McCarthy, Tom McRaet, Peter Menkhorst, Ren Millsom, David Milton, Clive Minton, Euan Moore, Deane Morgan, Alan Morris, John Mullins, Tim Murphy, Vicki Natt, John Newman, Mike Newman, David Niland, Gavin O'Brien, James O'Connor, Jo Oldland, Jan Olley, Kim Onton, Max O'Sullivan, Richard Owen, Bob Patterson, Priscilla Park, Lynn Pedler, Joy Pegler, Doug Phillips, Hugo Phillipps, Robyn Pickering, Chris Purnell, Rick Ressom, David Rohweder, Rosemary Payet, Robyn Pickering, Ivor Preston, Bianca Priest, Ken Read, Jim Reside, Danny Rogers, Ken Rogers, Dave Ryan, Toni Ryan, Dick Rule, Bill Russell, Bill Rutherford, Mike Schultz, Eric Sedgwick, Rob Shuckard, Bob Semmens, Marion Shaw, Paul Shelly, Andrew Silcocks, Donna Smithyman, Roger Standen, Simon Starr, Will Steele, Jonathon Stevenson, Ian Stewart, Phil Straw, Alan Stuart, Rob Tanner, Bryce Taylor, Ian Taylor, Susan Taylor, Margie Tiller, Kent Treloar, Chris Tzaros, Len Underwood, Dave Warne, Paul Wainwright, Bill Wakefield, Brian Walker, Doug Watkins, Toni Webster, Mike Weston, Gary Whale, Tom Wheller, Jim and Anthea Whitelaw, Bill and Evelyn Williams, Kelvin Williams, Eric Woehler, Jon Wren, Bill Wright, Boyd Wykes.
